# Supplementary material for: How much is “enough”? Considerations for functional connectivity reliability in pediatric naturalistic fMRI
Source: Imaging Neurosci (Camb). 2025 Aug 19;3:IMAG.a.117. doi: 10.1162/IMAG.a.117 (PMC12365692; doi:10.1162/IMAG.a.117)
Supplement: Supplementary Material [file IMAG.a.117_supp.pdf]

What was the name of the ancient lost city?

A) Papaya

B) Parapata

C) Pandora

Sleep Rating: **(check any that apply)**

- ☐ I felt tired
- ☐ I felt sleepy
- ☐ I had difficulty staying awake

How did Dora feel when Diego was leaving to move away?

A) Angry

B) Annoyed

A) Sad

What kind of toy did the boy take out of the box?

A) Dinosaur

B) Legos

C) Fire Truck

Sleep Rating: **(check any that apply)**

- ☐ I felt tired
- ☐ I felt sleepy
- ☐ I had difficulty staying awake

How did the rainbow sand video make you feel?

A) Sad

B) Calm

C) Worried

D) Other: _____________

What season is the forest walking video showing?

A) Winter

B) Fall

C) Nighttime

Sleep Rating: **(check any that apply)**

- ☐ I felt tired
- ☐ I felt sleepy
- ☐ I had difficulty staying awake

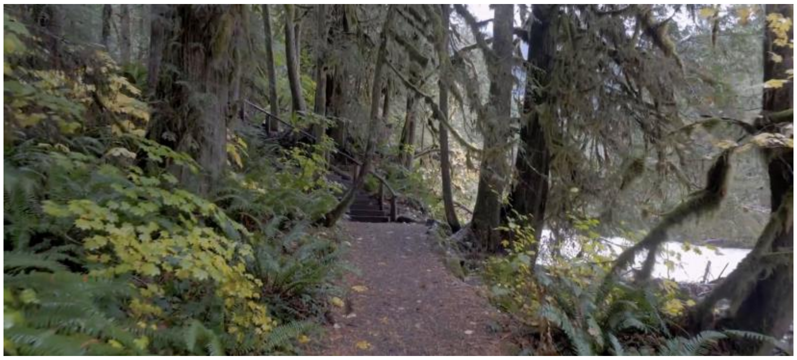

Supplemental Table 1: Example post-MRI survey

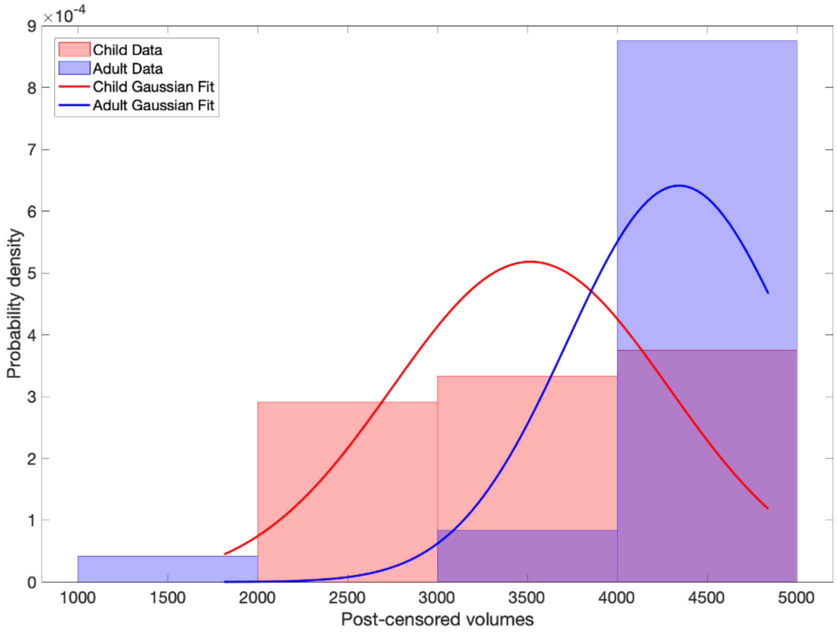

Supplemental Figure 1: Probability density distribution of post-censored data acquired from adults and children during naturalistic fMRI. Plotting the probability density across both groups, we observed an intersection of the curves at 3863 post-censored volumes. This point served as the threshold for classifying participants into the low-motion adults (LMA), low-motion children (LMC), and high-motion children (HMC) groups.

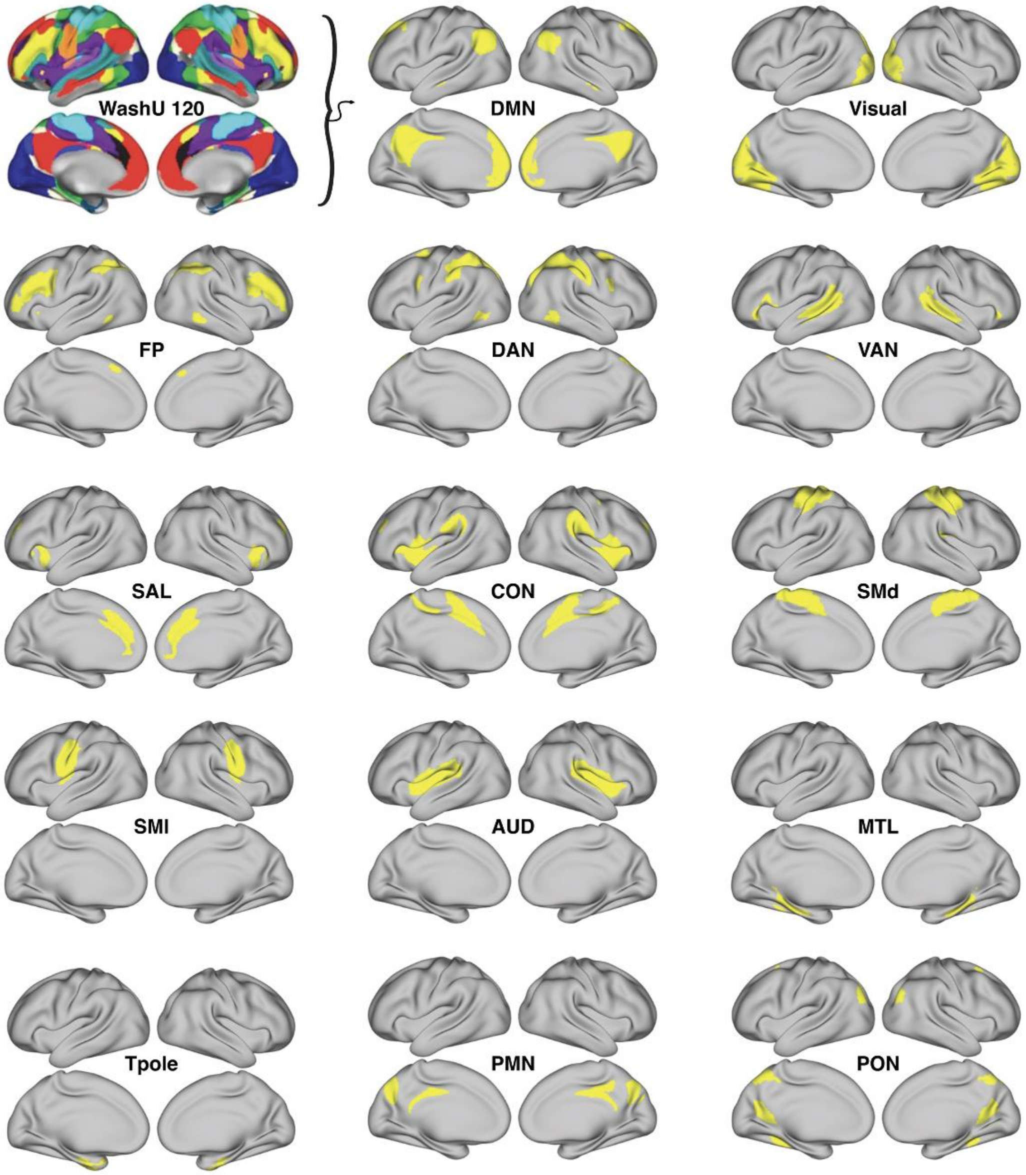

Supplemental Figure 2: 14 network templates binarized to the WashU 120 template using vertex time series data from the Midnight Scan Club dataset, processed in a previous study from our group (Rai et al., 2024).

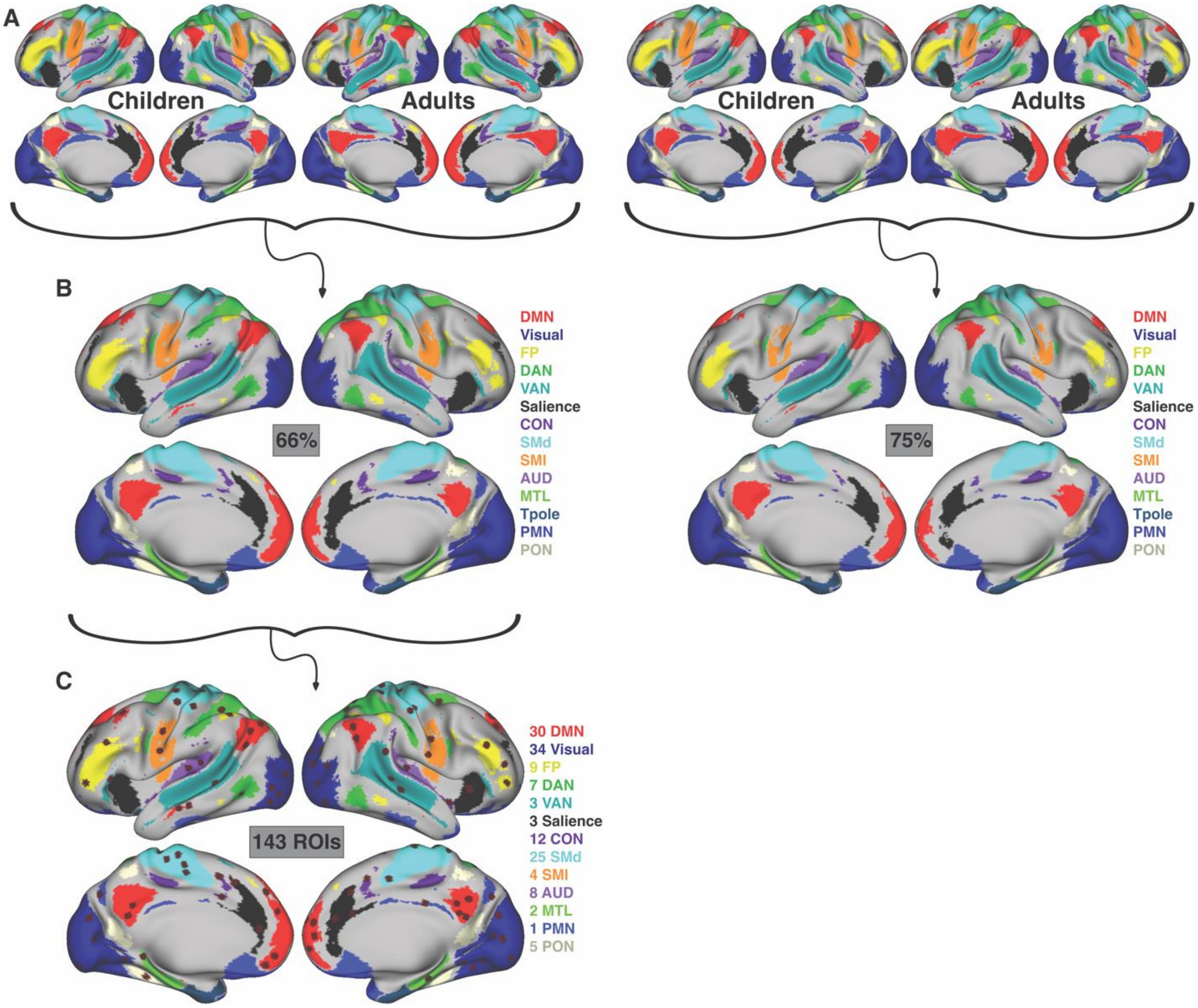

Supplemental Figure 3: 143 ROIs shown plotted on the combined adult and child group overlap maps under passive viewing conditions. A) Group network maps for adults and children using 66% and 75% thresholds. B) Overlap network maps between adults and children. C) The final overlap map with 143 ROIs used in the study.

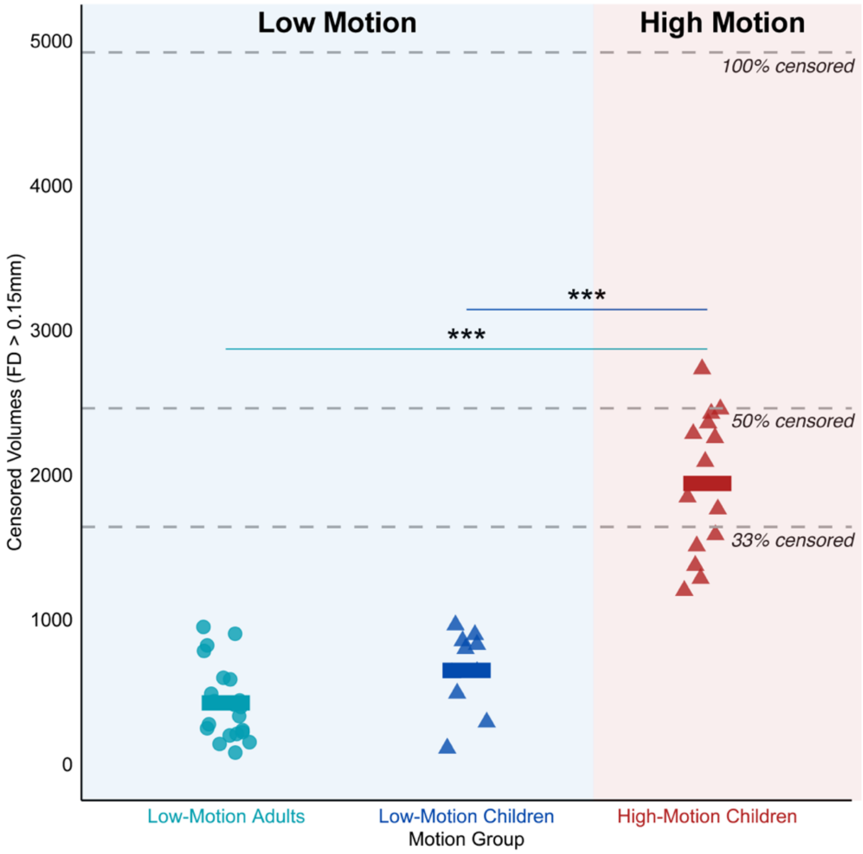

Supplemental Figure 4: Amount of data censored across all passive viewing conditions for each motion group. The number of censored volumes (FD>0.15 mm) were significantly different between the high-motion group (HMC, red triangles) and both the low-motion adults (LMA, light blue circles) and low-motion children (LMC, dark blue triangles). The low-motion adults did not significantly differ in censored volumes with the low-motion children (p-adjusted = 0.223). Dotted lines represent percent of volumes censored out of the 4920 total pre-censored volumes collected. Significance: *** p<0.001.

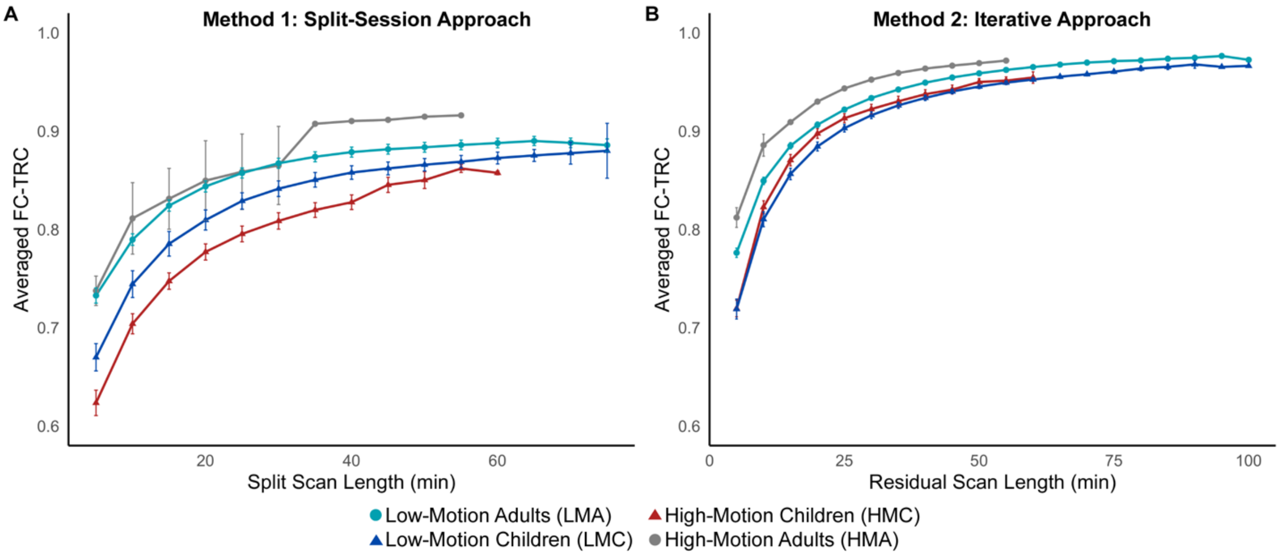

Supplemental Figure 5: Group averaged FC-TRC-by-scan duration curves combined across all viewing conditions. Averaging across participants in each motion group, both the split-session approach (A) and iterative approach (B) revealed differences between the low-motion adults (LMA, light blue circles) and both child groups. A) The split-session approach indicated that the low-motion children (LMC, dark blue triangles) attained higher FC-TRC values compared to the high-motion children (HMC, red triangles). Compared to (A), the iterative approach in (B) had a smaller gap in FC-TRC differences between both child groups and the low-motion adult group. The high-motion adult group (HMA, grey circles) included two participants that were excluded from further motion group analyses.

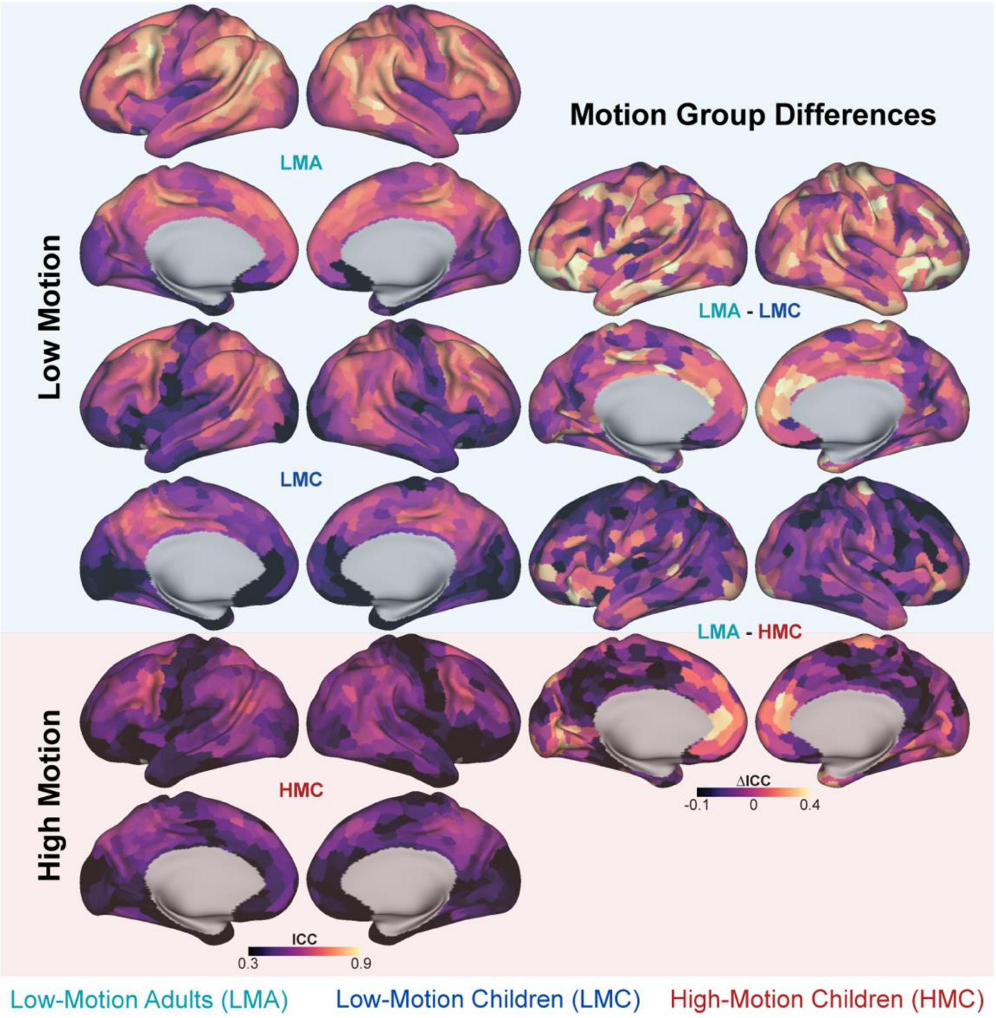

Supplemental Figure 6: Regional differences in Intraclass Correlation Coefficient (ICC) reliability across motion groups under passive viewing conditions. The low-motion adult (LMA) group exhibited greater overall ICC reliability with difference maps between groups shown in the right column. The largest difference observed was between the low-motion adults (LMA) and high-motion children (HMC) in frontal and temporal lobe regions. The Schaefer 1000 parcel 17-network atlas was used to parcellate brain regions.

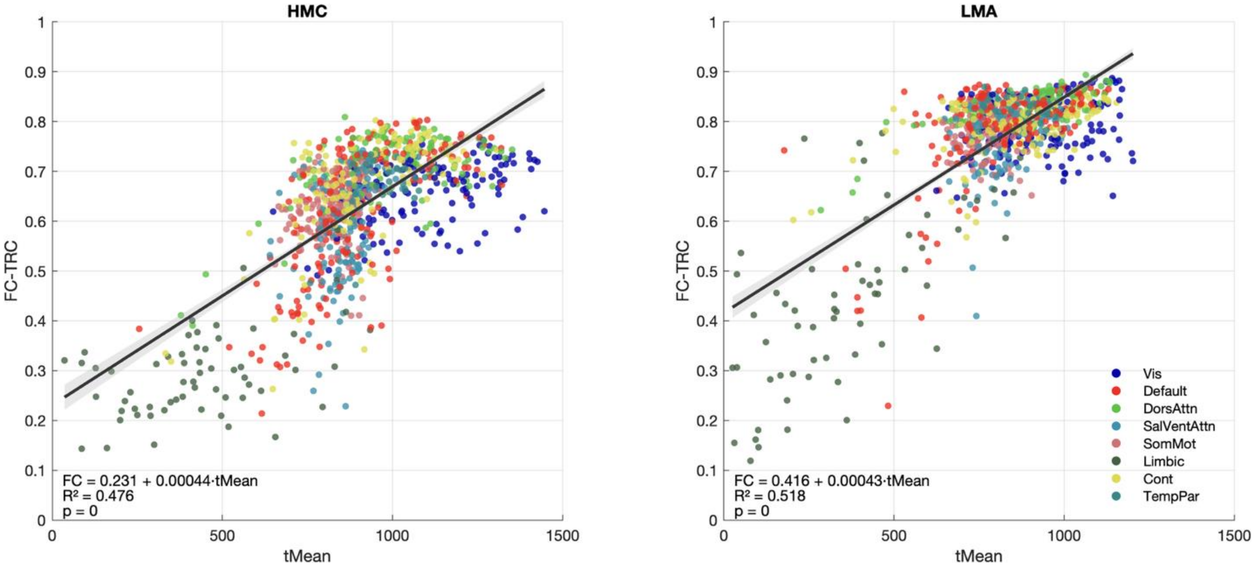

Supplemental Figure 7: Temporal mean signal (tMean) versus FC-TRC across 1000 parcels for both low-motion adults (LMA) and high-motion children (HMC). Parcels with higher tMean reach greater levels of FC reliability in both LMA and HMC groups. Each point represents a single parcel and is color-coded by its Schaefer network assignment. Linear regression lines and equations are shown on the plot.

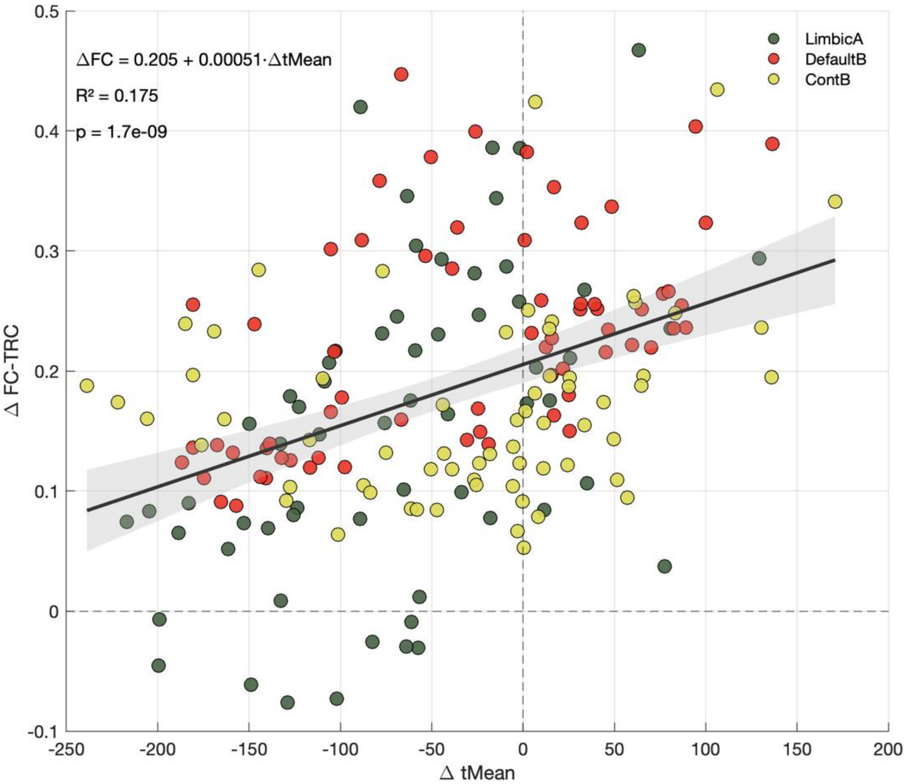

Supplemental Figure 8: Group differences in FC-TRC and temporal mean signal between low-motion adults and high-motion children. Parcel-wise differences in FC-TRC (LMA – HMC) and differences in tMean (LMA – HMC) are shown for parcels within the Limbic A, Default A, and Control B networks. Parcels with the largest positive group differences in FC-TRC also had the largest differences in tMean between LMA and HMC. Linear regression lines, equations, and R2 values are shown on the plot.

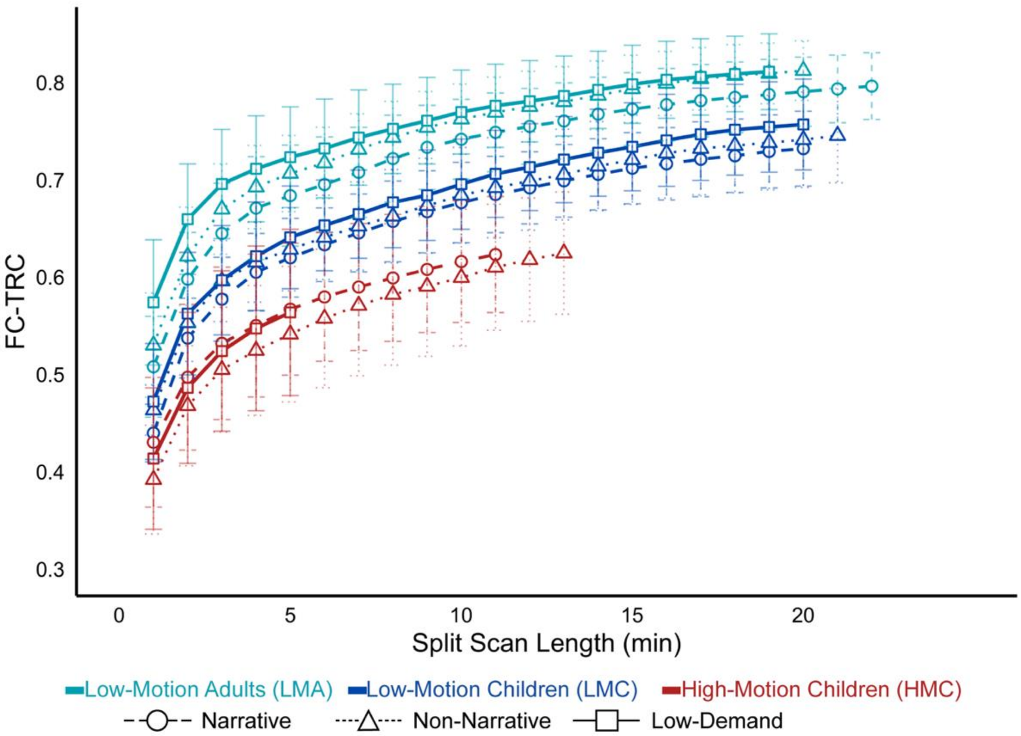

Supplemental Figure 9: Test-retest correlations (FC-TRC) curves across each viewing condition. The low-motion adults (LMA, light blue) showed higher FC-TRC values compared to both low-motion children (dark blue) and high-motion children (red) across all conditions. Across all motion groups, the low-demand condition, indicated by squares, achieved the highest FC-TRC, however, retained the least split-half data for the high-motion children (HMC) at 5 minutes.

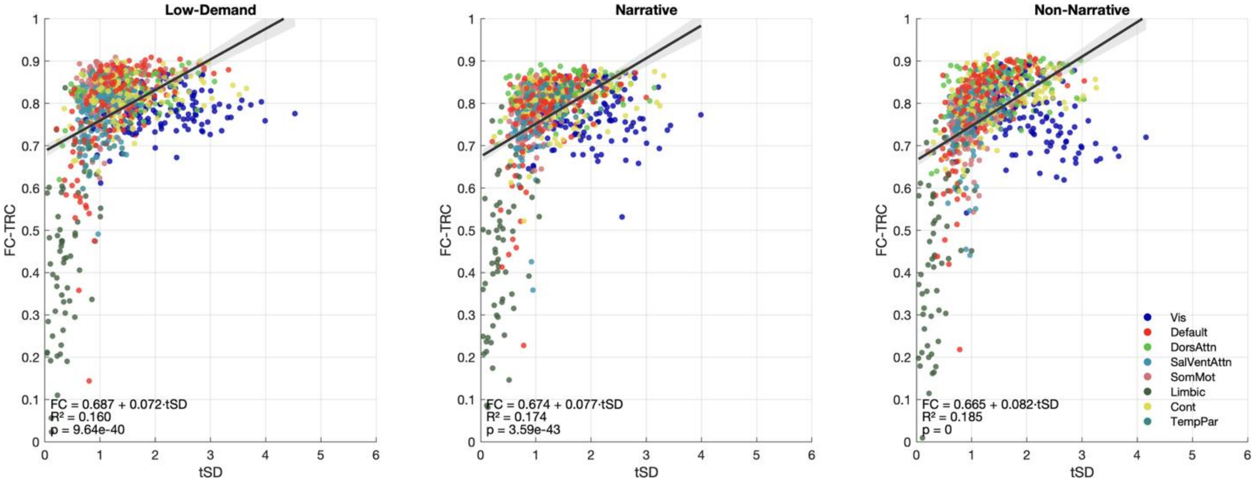

Supplemental Figure 10: Temporal standard deviation relates to FC-TRC across conditions. Using data from 8 exemplar low-motion adults during the three viewing conditions, we found a positive relationship between temporal standard deviation and FC-TRC. Each point represents a Schaefer atlas parcel, color-coded by network assignment. Linear regressions lines and are displayed on the plot.

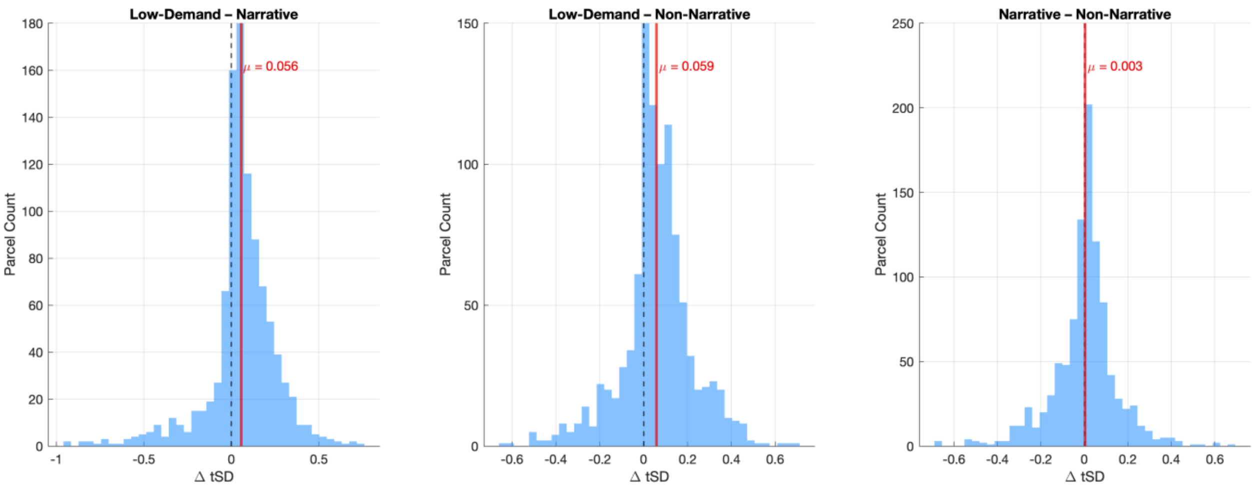

Supplemental Figure 11: Delta temporal standard deviation distribution across conditions. Parcel-wise distribution of delta tSD between pairwise conditions is shown across 8 exemplar low-motion adults. Mean differences in tSD were higher for comparisons with the low-demand condition, compared to between the narrative and non-narrative conditions. Red vertical lines denote the mean delta tSD across comparisons.

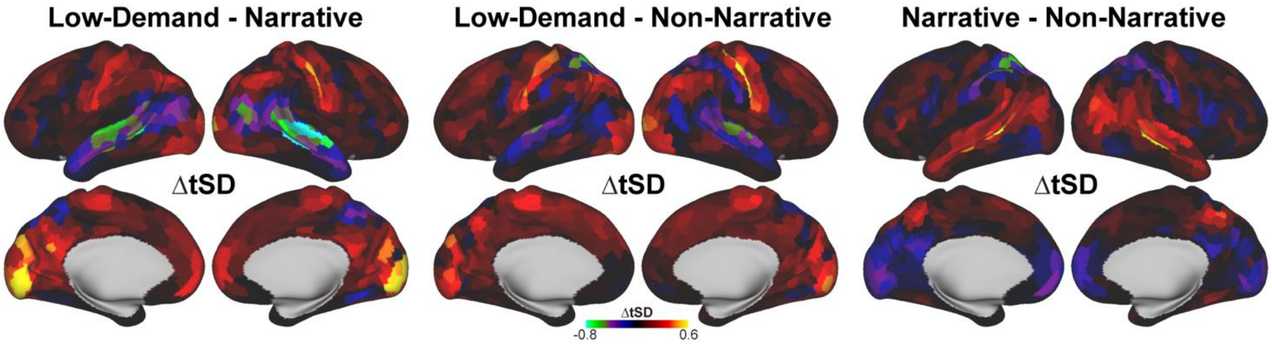

Supplemental Figure 12: Surface maps depict differences in temporal standard deviation between conditions. Using the 1000 Schaefer parcels across the cortical surface, differences were most prominent between low-demand and narrative conditions. Visual parcels showed the greatest increase in temporal standard deviation and superior temporal parcels showed decreases in tSD in the low-demand condition compared to the narrative condition. The smallest differences in tSD are visible between narrative and non-narrative conditions.
